# How Gender Shapes Parent-Adult Child Consistency of Reporting Problems in Children’s Lives

**DOI:** 10.1093/geroni/igaf122.3296

**Published:** 2025-12-31

**Authors:** Tamrah Verbanac, Destiny Ogle, Megan Gilligan, J Jill Suitor

**Affiliations:** Purdue University, West Lafayette, Indiana, United States; Purdue University, West Lafayette, Indiana, United States; University of Missouri, Columbia, Missouri, United States; Purdue University, West Lafayette, Indiana, United States

## Abstract

Despite both parents’ and adult children’s declarations that they have an accurate picture of one another’s feelings and behaviors, research that has collected data separately from both members of parent-adult child dyads has shown that there are typically substantial discrepancies in their reports. One area in which such discrepancies may be of particular concern is adult children’s problems. Although parents are often a central source of practical support when adult children face crises, even in midlife, providing adequate support at the most advantageous time may be hindered by a lack of information sharing between the generations. In the present paper, we compare children’s reports of their current and recent problems with parents’ reports of their children’s problems, using data collected from mothers and fathers (aged ∼70) and at least one of their adult children (aged ∼40) in 85 families as part of the Within-Family Differences Study-I. Using a combination of quantitative and qualitative data, we explore how consistency is reporting is shaped by a combination of the type and timing of the problem, and the gender of both the adult child and the parent. Preliminary findings suggest that consistency is highest between mothers’ and daughters’ reports, and lowest between fathers and daughters. Consistency is also greater when problem are current and are less likely to potentially “reflect badly” on either the child or the parent (e.g., illnesses and non-culpable injuries). These findings can be helpful to clinicians developing strategies to increase communication across generational ties, particularly in times of crisis.

